# Neuralgic amyotrophy with multiple hourglass-like constrictions of anterior interosseous nerve: a case report

**DOI:** 10.3389/fneur.2024.1306264

**Published:** 2024-01-29

**Authors:** Fangling Shi, Xiaoling Zhou, Xueyuan Li

**Affiliations:** Department of Hand Surgery, Ningbo No.6 Hospital, Ningbo, China

**Keywords:** neuralgic amyotrophy, Parsonage-Turner syndrome, brachial neuritis, surgery, neurolysis

## Abstract

Hourglass-like constrictions (HLCs) of peripheral nerves in the upper extremity were a rare form of neuralgic amyotrophy, often characterized by the sudden onset of pain in the shoulder or arm, followed by muscle weakness and amyotrophy, with limited sensory involvement. We present a case of multiple HLCs of the anterior interosseous nerve (AIN) in a 22-year-old female with left upper arm pain, finger numbness, and limited activity for 1 month. Physical examination showed weakness of the left index flexor digitorum profundus and flexor pollicis longus, with mild hypoesthesia in the first three fingers and the radial half of the ring finger. Electromyography suggested a median nerve (mainly AIN) lesion. Ultrasonographic imaging of the median nerve shows AIN bundle swelling and multiple HLCs at left upper arm. Despite conservative treatment, which included 15 days of steroid pulse therapy, Etoricoxib, and oral mecobalamin, the patient still complained of extreme pain at night without relief of any symptoms. Operation was recommended for this patient with thorough concerns of surgical advantages and disadvantages. During surgery, a total of 7 HLCs were found in her median nerve along and above the elbow joint. Only Interfascicular neurolysis was performed because the nerve constrictions were still in the early stage. The pain was almost relieved the next day. One month after surgery, she could bend her thumb and index fingers, although they were still weak. 4 months after the surgery, she was able to bend affected fingers, with muscle strength M3 level. At the same time, her fingers had fewer numbness symptoms. There was still controversy regarding treatment strategy; however, early diagnosis and surgical treatment for nerve HLCs might be a better choice to promote nerve recovery.

## Introduction

Neuralgic amyotrophy, also known as brachial neuritis or Parsonage-Turner syndrome, is characterized by the sudden onset of pain in the shoulder or arm, followed by muscle weakness and amyotrophy, with limited sensory involvement (1–3). The etiology of neuralgic amyotrophy is still unclear. The pathology points toward certain autoimmune processes, induced by a viral infection or activated in an immunosuppressed state, or an allergic mechanism, leading to inflammation of selected peripheral nerves (4, 5).

With the application of high-resolution peripheral nerve imaging including, ultrasound and MRI, the presence of HLCs of involved nerves or nerve fascicles is increasingly recognized in patients with neuralgic amyotrophy (6), and patients with HLCs usually have poor recovery with residual symptoms or paresis (7). Ultrasound has the advantage of being noninvasive and cost-effective, providing important value in monitoring the progress of affected nerves (6, 8).

We achieved good clinical recovery in a neuralgic amyotrophy patient with multiple HLCs in the AIN by early surgical intervention. We discussed the early diagnosis and treatment strategy based on this special case. Written consent for publication of this case report was obtained from the patient herself.

## Case report

### History and examination

**Figure 1:**
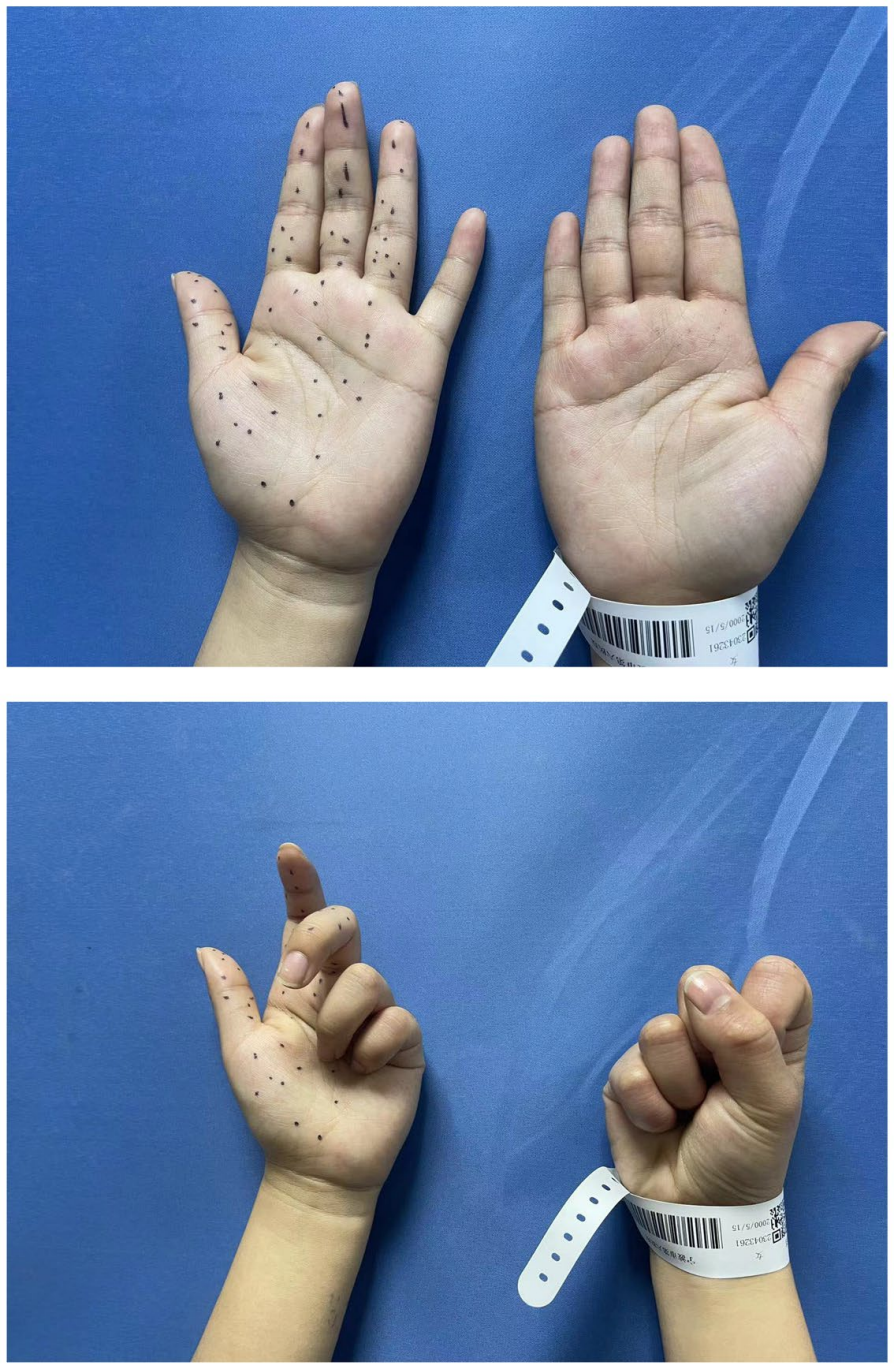
Preoperative physical examination showed the strength of the left flexor pollicis longus and index flexor digitorum profundus was MRC 2, with mild hyperesthesia in 1–4 digits.

A 22-year-old woman complained of extreme pain in her left upper arm and numbness in her fingers after carrying heavy acrylic plates. There was no family history of nervous system or musculoskeletal disease. Initially, she was unaware of the severity of the condition. She chose to stay at home and take a rest. However, her pain and weakness showed no signs of recovery, with obvious flexion weakness of her thumb and index finger within 2 weeks. Half a month after the onset of the disease, ultrasound examination at a local hospital revealed edema of the median nerve accompanied by constrictions. One month following the onset, she came to our outpatient clinic. The patient presented with a forced left elbow flexion position due to severe pain. Physical examination showed a severe paresis of the left flexor pollicis longus and index flexor digitorum profundus muscles (strength 2 on the Medical Research Council (MRC) scale), a moderate paresis for the middle flexor digitorum profundus (MRC 3), biceps brachii, flexor carpi radialis, and abductor pollicis brevis muscles (MRC 3–4), with mild hypesthesia first three fingers and the radial half of the ring finger (Figure 1). The rest of muscle strength, tendon reflexes, Tinel’s sign and neck examination were unremarkable.

At first, we adopted conservative treatment for the patient including 2 weeks of steroid pulse therapy (1 mg/kg), etoricoxib and oral mecobalamin. However, the patient still complained of extreme pain described as shooting and drilling at night. Therefore, electromyography (EMG) and ultrasound examination were performed when she came to the clinic again.

The EMG results showed that the abductor pollicis brevis, flexor pollicis longus, flexor carpi radialis, and biceps brachii exhibited fibrillation potentials and positive sharp waves, indicating AIN and musculocutaneous nerve both involvement. The conduction velocity of Median nerve was 53 m/s and the compound muscle action potential (cMAP) of the biceps muscle, as shown in EMG, was 10.5 mV (Table 1).

**Table 1:** Patient’s electromyography results.

Muscle	Fibrillation potentials	Positive sharp waves	cMAP	Lat
Abductor pollicis brevis	➕	➕	1.3	4.3
Flexor pollicis longus	➕	➕	—	—
Flexor carpi radialis	➕	➕		
Biceps brachii	➕	➕	10.5	10.8

Detected nerves	cMAP(Left)	Lat(Left)	cMAP(Right)	Lat(Right)
Lateral antebrachial cutaneous nerve	42.3	1.4	40.1	1.3
Superficial radial nerve	58.7	1.7	—	—
Median nerve	18.4	3.1	43.1	3.1

**Figure 2:**
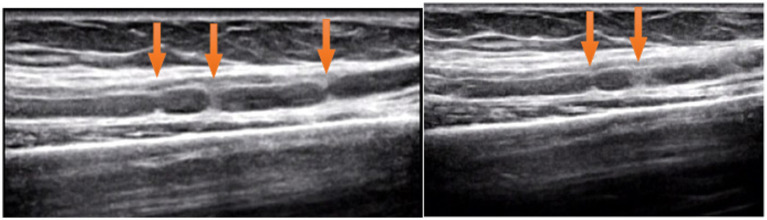
The longitudinal ultrasound image shows multiple HLCs of AIN (arrow).

Ultrasound examination of the median nerve showed nerve bundle swelling and multiple HLCs of left upper arm (Figure 2). Magnetic resonance imaging was not performed.

### Surgery

The Median nerve was exposed through 2 incisions in front of the elbow joint. Inspection of the median nerve revealed that nerve fascicles (mainly AIN) were partially swollen and hardened due to inflammatory thickening of the epineurium proximal to the medial epicondyle.

**Figure 3:**
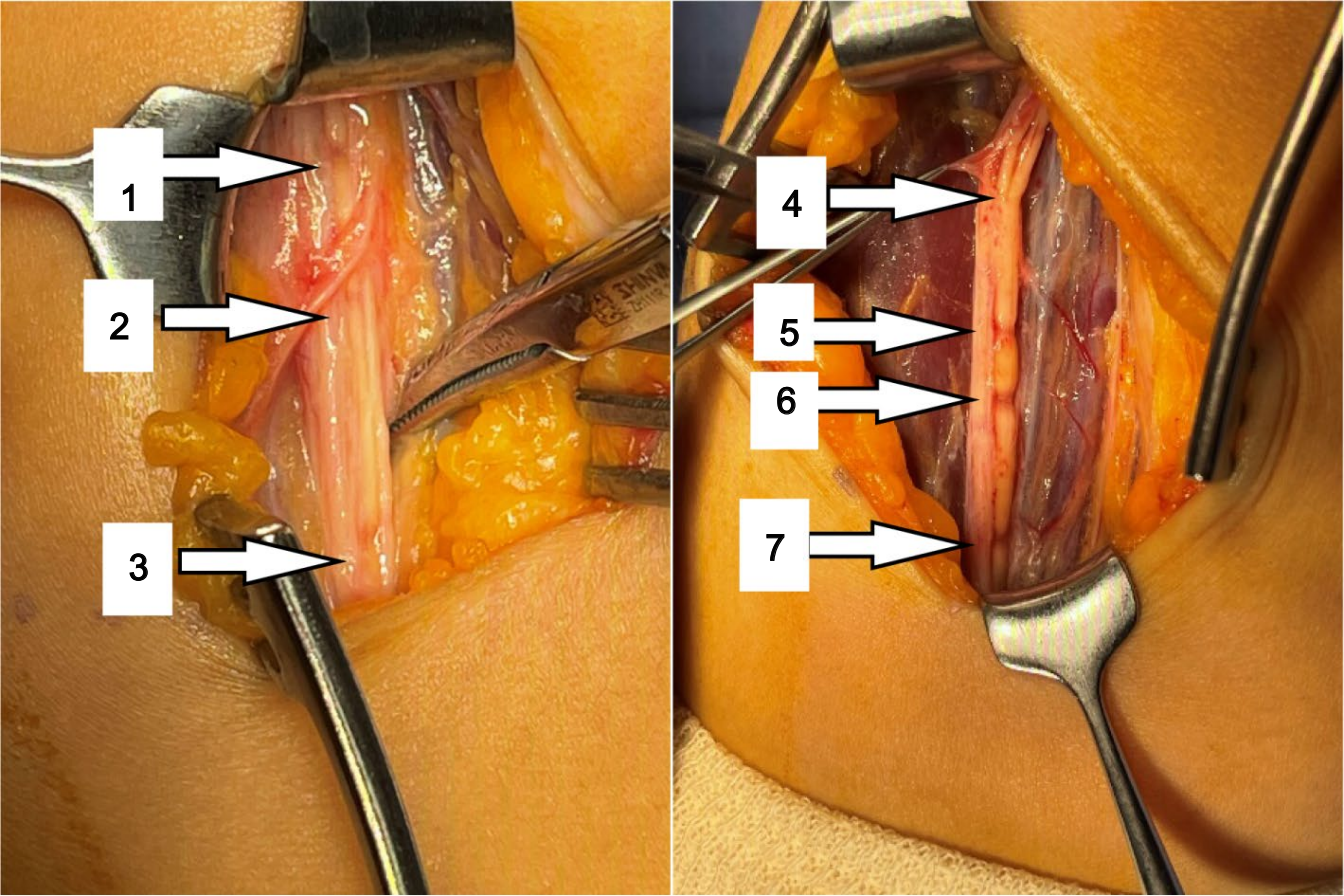
Multiple HLCs had been observed in the affected AIN during the operation (arrow).

After the thickened epineurium had been opened, multiple HLCs lesions along with AIN bundle were exposed. One of the fascicles showed 4 HLCs and other fascicles showed 3 HLCs (Figure 3).

During surgery, a total of 7 HLCs were found at 10.5, 9.5, 9.0, 6.0, 2.0, 1.5 and 0 cm proximal to the medial epicondyle (Figure 3) in her AIN bundle and without any visible source of external compression. During surgical intervention, nerve epineurium and part of the interfascicular nerve were released and electrical stimulation was performed on the median nerve. A total of 1 mL Compound Betamethasone was injected underneath the affected AIN epineurium.

### Postoperative course

After surgery, the left elbow was immobilized at 135° position with splint for 2 weeks. The next day, the patient’s pain was almost relieved. One month after surgery, she was able to bend her thumb and index fingers actively, although still weak (MRC 2). 4 months after the surgery, she was able to bend her thumb and index finger (MRC 3). At the same time, her fingers were less numbed.

## Discussion

The structural pathology of HLCs has been described in numerous cases of neuralgic amyotrophy, affecting various nerves including AIN, PIN, musculocutaneous nerve, suprascapular nerve and posterior cord (9). Recent progress in research on high-resolution imaging has effectively improved the understanding and early diagnosis of neuralgic amyotrophy (4, 6, 8). Moreover, Peripheral nerve surgery is gradually recognized as a routine treatment for neuralgic amyotrophy with HLCs (4, 5, 7, 10). In this case, we diagnosed AIN nerve constrictions early using ultrasound and performed surgical procedures after 2 weeks of conservative treatment, avoiding further progression of nerve constriction.

For decades, the diagnosis of neuralgic amyotrophy has been mainly based on specific symptoms, such as acute intense pain around the shoulder girdle followed by muscle weakness and amyotrophy (3, 4, 11). In the early stage of neuralgic amyotrophy, EMG may indicate that 2 or more nerves are involved. The EMG characteristics in this case indicated nerve axonal injury and compared with sensory nerves, mainly the axons of the motor nerves were damaged. The clinical features of this case include severe pain and acute muscle weakness, which are consistent with the clinical diagnosis of neuralgic amyotrophy. One month after the onset of the disease, electromyography showed that AIN was severely damaged, with partial axonal damage of the musculocutaneous nerve.

High-resolution peripheral nerve imaging such as MRI and ultrasound technology are considered valuable tools for the diagnosis of neuralgic amyotrophy based on the pathologic basis of HLC (2, 12, 13). Arányi et al. reported abnormal ultrasound findings in 74% of neuralgic amyotrophy patients and classified abnormalities as swelling without constriction, swelling with incomplete constriction, swelling with complete constriction, and fascicular entwinement (14, 15). However, there are few reports of HLC in patients with early-stage neuralgic amyotrophy. Paolo et al. described 39 patients with neuralgic amyotrophy, of which 29 had the presence of HLCs detected by ultrasound within 1 month of onset and recommended using ultrasound as the first-line complementary tool within the first 2 weeks after symptom onset (6). In this case, at 2 weeks of onset, ultrasound examination revealed edema and HLCs in the patient’s AIN, and at 4 weeks of onset, there were no signs of improvement in the AIN. Ultrasonography showed that the AIN above the elbow joint had obvious swelling with multiple HLCs.

Neuralgic amyotrophy was previously considered a rare self-limited disease with good prognosis (16). Recent publications suggested that the incidence of neuralgic amyotrophy was severely underestimated, and its actual incidence rate is 1/1000 per year (17). Treatment includes early administration of corticosteroids, appropriate pain management, and physiotherapy. Pan et al. explored the nerves of patients with persistent palsy and no signs of clinical recovery and found that HLC lesion in the nerve is the pathological basis of brachial neuritis (7). On the basis of poor clinical recovery and HLCs, Pan et al. consider surgical intervention as a treatment option to deal with those patients who do not respond to conservative treatment after several months (7).

However, there was still controversy regarding treatment strategy and timing of surgical intervention. In this particular case, operation was recommended with thorough concerns about surgical advantages and disadvantages as there is no significant improvement after conservative treatment, and more importantly, the ultrasound confirmed the presence of constriction in the nerve bundles which usually predicts poor recovery. The intraoperative findings highly coincided with the results of preoperative ultrasound. Interfascicular neurolysis and fascicle stimulation were performed because the nerve constrictions were still in the early stage and the nerve constrictions were incomplete, except the 6th and 7th constrictions were 80% constriction. After interfascicular neurolysis, the nerve structure was still continuous and acceptable.

In this case, even though we discovered HLCs in AIN early on, the patient still recovered poorly after conservative treatment. However, we believed that early diagnosis is important to allow prompt neurolysis surgery that was beneficial for the patient’s functional recovery. During neurolysis, the pressure inside the perineurium and between the interfascicular nerve bundles was released. Local Betamethasone was injected beneath the perineurium to prevent further edema. Fortunately, the patient’s symptoms were relieved in a short period.

Kim et al. (18) speculated that delay of the surgery, age of the patient, and method of surgical treatment indicated poor prognosis. Therefore, if there is a clear diagnosis indicating the presence of HLCs and poor recovery after conservative treatment, surgical treatment is the best choice to avoid further narrowing of the affected nerves and promote functional recovery.

The selection of surgical methods is based on the results of intraoperative nerve stimulation, personal experience of the surgeon, and the basis of the degrees of constriction found at surgical exploration (7). Severity of constriction was also defined by Gstoettner according to the percentage of nerve/fascicle thinning: ≤25% thinning was classified as mild, 25–75% as moderate and ≥ 75% as severe constriction (4). In complete constriction cases, interfascicular neurolysis only was not recommended, and patients should undergo neurorrhaphy. In this case, the 6th and 7th contractions in Figure 3 are severe, but we believe that functional recovery was still possible by neurolysis since the constriction was not complete and this case was still in its early stages. The prognosis of this patient confirmed our hypothesis.

For neuralgic amyotrophy, early diagnosis and surgical treatment for nerve hourglass-like constrictions might be a better choice to promote nerve recovery speed. Neurolysis helps reduce pressure in the nerves, restore muscle function, and shorten the course of the disease in the early stages.

## Data availability statement

The original contributions presented in the study are included in the article/supplementary material, further inquiries can be directed to the corresponding author/s.

## Ethics statement

Ethical approval was not required for the study involving humans in accordance with the local legislation and institutional requirements. Written informed consent to participate in this study was not required from the participants or the participants’ legal guardians/next of kin in accordance with the national legislation and the institutional requirements. Written informed consent was obtained from the individual(s) for the publication of any potentially identifiable images or data included in this article.

## Author contributions

FS: Data curation, Software, Writing – original draft. XZ: Methodology, Supervision, Writing – review & editing. XL: Project administration, Supervision, Writing – original draft, Writing – review & editing.
